# Designing programmes of physical activity through sport: learning from a widening participation intervention, ‘City of Football’

**DOI:** 10.1186/s12889-018-6049-6

**Published:** 2018-09-26

**Authors:** Stephen Zwolinsky, Nicola Kime, Andy Pringle, Paul Widdop, Jim McKenna

**Affiliations:** Carnegie School of Sport, Centre for Active Lifestyles, Leeds Beckett University, Leeds, UK

**Keywords:** Physical activity, Clustering, Network analysis, Behaviour change

## Abstract

**Background:**

Implementation profoundly influences how well new audiences engage with sport-based physical activity programmes. Recognising that effective implementation relies on concurrently generating supportive contexts, systems and networks for the least engaged ‘target’ groups; this paper aims to address what underpins children’s (non) engagement with football-based physical activity.

**Methods:**

An observational research design, using a non-probability sample of *N* = 594 primary and secondary schoolchildren assessed outcomes of a three-year ‘City of Football’ (CoF) programme. Pupils self-reported football participation, personal friendship networks and exposure to six concurrent sources of influence (SoI). A 2-step hierarchical cluster analysis and univariate analyses assessed between-cluster differences.

**Results:**

Girls played football least regularly (χ^2^ [4] = 86.722, *p* = 0.000). Overall, participation was significantly associated with personal networks engaged in football. Boys’ personal networks were more stable and structurally effective. Football participation was also positively and linearly association with SoI scores. Girls and pupils with no personal networks around football reported the lowest SoI scores. Three clusters emerged, dominated by social network influences. The *Traditional Market* (*n* = 157, 27.7%) comprised 81.7% boys; they regularly played football, had the most effective network structure and scored highly across all six domains of SoI. The *Sporadically Engaging Socialisers* (*n* = 190, 33.5%) comprised 52.9% girls who rarely played football, reported low SoI scores and an inferior network structure. In the *Disconnected* cluster (*n* = 220, 38.8%), 59.3% were non-footballing girls who reported the lowest motivation and ability SoI scores; and no personal networks engaged in football.

**Conclusions:**

This study reveals new insights about the primacy of social network effects for engaging children in football-based physical activity programmes. With little or no attention to these social-oriented issues, such interventions will struggle to attract ‘target’ children, but will readily engage already well-connected, experienced football-playing boys. The challenge for drawing non-footballing children into football-based interventions lies with engaging children – especially girls - whose social networks are not football-focused, while they also find football neither personally motivating nor easy to do.

## Background

Optimising population physical activity levels - including participation rates in exercise and sport – is likely to be a key priority for all public health systems focused on efficiency and sustainability [1]. In part, this may be due to the extensive research and evidence base confirming the profound physiological, psychological and social health effects arising from sustained, enjoyable engagement with physical activity [2–5]. Yet, the challenge for public health and sporting organisations no longer solely lies with generating ever more reasons for people to participate. Instead, the pursuit is for effective intervention design and implementation that generates supportive contexts and networks sustaining engagement among all groups, especially the least active [6]. This signals an important shift away from being concerned with ‘why’ to intervene, to focus on understanding ‘how’ to intervene, especially among non-responding groups.

To generate lasting, significant and autocatalytic change, simultaneously tapping into essential behavioural levers is a prerequisite for dealing with any underlying unhealthy practices [7]. To tackle the insufficient proportion of boys and girls meeting the physical activity guidelines both nationally [8] and internationally [9], this concept becomes increasingly important. At present, less than 25% of boys and 20% of girls are sufficiently active for health in the UK [10]. Although girls are typically less active compared to boys [11], and demonstrate alarming declines in activity before and during the transition to adolescence [12], declines in achieving government guidelines are now comparatively greater among boys compared to girls between the ages of 13–15 years [13]. Furthermore, as the reach of organisational sport in children continues to stagnate [14], more attention needs to focus on approaches that contribute to improved public health. Nevertheless, outcomes from sport policies that champion inclusivity fail to evidence any meaningful impact [15]. Moreover, in the UK, sport funding and policy often excludes large proportions of the population, who are simultaneously inactive and show little interest in pursuing eliteness [16]. Given the growing evidence surrounding effective theoretical frameworks and systems based approaches [7, 17], the on-going lack of inclusivity suggest that practice within some sports lags behind the evidence base in terms of programme design, policy and funding strategies.

Attempting to discover a single cause, and therefore enact a single solution, for any complex problem is common [18] but often a signal of programme irrelevance. Better solutions normally emerge by viewing problems as complex, and by changing the systems that surround people so they become energised and capable of functioning within those systems. This approach can address influential factors left unaffected by typical design and implementation. Behavioural systems approaches [17] point to the powerful impact of concurrently addressing the underlying influence on behaviour [7, 19], including bundles of socially constructed practices [20, 21]. Behavioural systems predict that regular positive experiences promote the motivation and ability to adopt a behaviour, whereas negative experiences can lower motivation and perceived ability to participate [17]. In this understanding, individual decisions and actions affect those of peers and vice versa [22, 23]. That is, no individual’s behaviour exists outside of social context; purposive actions are instead embedded in concrete on-going systems of social relations [24]. This understanding, supplemented by recognition of children’s reliance on emotion-based learning, explains why programmes aiming to help young people often focus on a social-emotional lens [25].

These are useful concepts for any sporting organisation and sports policymakers seeking to boost grassroots participation among traditionally unresponsive groups. Nevertheless, their operationalisation is likely to be problematic. For example, few sport-based programmes attempt to influence social-economic factors, even though they may powerfully influence local environments, social interaction and individual choice [26]. Worse, left unaddressed, these factors may inadvertently exacerbate the existing marginalisation of non-participating groups [27]. Further, while increased social interaction and access to resources embedded in networks - social capital - [28, 29] is thought to be among the greatest return that sports participation can generate [26], the principle interactions and networks experienced by marginalised groups are often levers for *non*-participation. There can be contagion by conversation, whereby people who talk together, participate - or not - together [30]. For that reason, different clusters of people are likely to react distinctively to social influence, meaning these groups will often require tailored individual and system level stimuli [23].

This paper aims to strengthen understanding of the behavioural system underpinning children’s engagement with a citywide football initiative seeking to widen participation. Our approach assesses behaviour at multiple levels of influence [19], (i) Personal – an individual’s capability and desire, (ii) Social – how others enable and encourage behaviour, and (iii) Structural – how systems promote and facilitate engagement [7]. Moreover, this paper aims to shed light on how children’s social networks influence participation and examine how structural clustering techniques can be used as a means to develop better ways of generating engagement among non-participating groups. We hypothesise that non-participating clusters of children will be characterised by weak influences for supporting football-specific behaviours and forming personal friendship networks.

## Methods

### City of football (CoF) overview

Football is England’s most popular team sport. Nevertheless, governing organisations understand that there is real cross sector benefit, not to mention capacity to grow the game. In 2014, the city of Nottingham received £1.6 million of funding from Sport England to deliver CoF [31]. The successful bid incorporated a 3-year pilot study and digital platform aiming to get people from non-participating groups in the city regularly playing football (http://www.city-of-football.uk/). In part, Nottingham’s commitment to promote the sport at the grassroots level was a reason for the bids success. CoF planned to activate private, public and voluntary sector partners from both inside and outside the traditional ‘football family’.

Nottingham CoF sought to shift the profile of people playing, coaching and volunteering in football across the city by reaching inactive individuals, especially children, girls and people from black and minority ethnic backgrounds. These groups were targeted as the greatest health benefits arise from inactive groups becoming more active [32]. Further, participation rates by ethnicity confirm the lowest levels of sports participation among BAME groups [33]. What is more, females are almost 12 times less likely to play football once a month or more compared to males, while only 15% of all female participants come from BAME backgrounds [34]. Finally, the FA’s strategy for growing the game centres on women and girls’ football [35].

CoF offered a range of physical actives and outreach sessions that utilised general health promotion information and messages. For example, activities seeking to encourage mass participation included a monthly CoF programme on ‘Notts TV’, a workforce strategy to train the next generation of sports coaches, ‘ParkLives’ summer events involving football with families, and football rocks, a music themed tournament at Notts County’s stadium. In relation to women and girls, CoF established girl’s-only football nights and summer camps, mums’ football and women’s veteran sessions. For children, activities included ‘Socatots’ and a school transition project fusing football and dance to encourage participation. CoF was also central to the development of Malawi football sessions, the south Asian football consortium and the Ummah (community) league at Forest sports zone. There were also a range of activities focussing on disability football in local schools and community centres, including amputee football, autism awareness and ‘inclusive’ football sessions.

In 2016, CoF commissioned an evaluation to determine the on-going influence of football among schoolchildren with a view to growing the game among non-participating groups. Nested within the CoF pilot study, this research utilised a non-probability sample of participants exposed to the CoF programme.

### Guiding framework

Intervention success is influenced by design, implementation and the host system [6]. Therefore, even for simple initiatives, interactions resulting from the key agents in these systems can be highly complex. Understanding how they work in practice is vital to building a functional evidence base that can enhance practicality and the likelihood of translating the research findings into practice. Nevertheless, practitioners have few, if any, methods to assess the integration and implementation of physical activity, exercise and sport into routine practice. Equally, evaluators and researchers need designs and practical solutions to assess influence across the necessary constituent parts. The ‘influencer’ framework adopted by this study, facilitates the needs of both these groups.

The ‘influencer’ framework proposes that behaviour is influenced by changing motivation and ability across three layers, leaving six areas of influence (personal motivation, personal ability, social motivation, social ability, system motivation and system ability) [7]. It seeks to clarify measurable results, find preferred approaches, while confirming the scale of influence of its six respective domains. Importantly, the framework relies on the concept of ‘over determining change’, which entails on-going prevention of relapse and/or dropout. Fundamentally, because of the risk of failing engagement, programme success relies on sustaining the most powerful individual, combined and sequential influences on behaviour, even when they may not seem necessary [19].

### Measures/data capture

Following institutional research ethics clearance and consent from participating head teachers, pupils participated in the research from December 2016 to March 2017. Prior to formally engaging the research process, having read an information sheet, consent to participate was obtained through written informed consent and parental (/guardian) assent for all participants under the age of 16. Data capture took place at participating sites through a brief 2-page survey. Eleven schools from three local CoF areas and Gamecity (the National videogame arcade) - identified by CoF staff to provide a stratified sample of pupils - comprised the data collection sites.

To address the research questions, pupils reported how frequently they played football inside and outside school on a five-point scale [36]. The survey also included a modified 12-item Sources of Influence (SoI) questionnaire to assess perspectives on motivation and competencies across three powerful behavioural domains [7]. Two questions addressed each domain, scoring responses 1 (Strongly Disagree) to 5 (Strongly Agree). SoI items have positive phrasing, meaning that high scores indicated strong coverage of a theme; these are scored (i) individually, (ii) socially, (iii) structurally and (iv) overall.

#### Ego network analysis

Ego network analyses centre upon connections around a particular node or ego (i.e. the connections within a network surrounding a person of interest) [37]; these can be used as a proxy for a level of personal networks. Each ego network includes that node (the actor, in this case the survey respondent), and all other nodes tied to it - often called ‘alters’. Ties between the ego and alters are then usually determined by the research question [38]. This connection can be based on friendship, trust, experience, or whatever is flowing through the network from node to node. Networks can intersect social circles, which is important, as most people interact and form ties across numerous and distinct domains [39]. These intersections are simpler to determine via ego-net analysis as the individual is the focus of the research; individuals may nominate alters from each of the social worlds in which they are engaged [37].

Study questions centred on exploring the football networks for participating schoolchildren (i.e., the ego). Six key network features were calculated. These were; (i) network size - the number of nodes and the ego, (ii) directed ties - connections detected among all the nodes in the ego network, (iii) ordered pairs - the maximum number of directed ties in each network, (iv) network density - the number of ties divided by the number of pairs, (v) effective size - the number of alters minus the average number of ties with each alter, and (vi) network efficiency - the effective size of each network by size.

#### Cluster analysis

Multivariate cluster analysis aims to identify homogenous groups according to shared characteristics [40]. This study used a two-step hierarchical cluster analysis with a log-likelihood distance measure to reflect both the sample size and the categorical and continuous inputs. Initial analysis forms pre-clusters, reducing the size of the matrix that contains distances between all possible pairs of cases. Then, cases merge with other pre-clusters, or form new ones. When this process is complete, all cases in the same pre-cluster are treated as a single entity. The second step uses a hierarchical algorithm to generate clusters from the pre-clusters to explore a range of likely solutions. Researchers made no assumptions about the number of clusters and membership to them, prior to the analysis. The final number of clusters was derived from the Schwarz Bayesian Criterion [41, 42]. This procedure also indicates predictor importance (PI) for the construction of each cluster.

The final cluster analysis identified five predictors of football engagement, (i) Social networks around football (PI 1.00), (ii) Playing football outside school (PI 0.77), (iii) Total SoI motivation score (PI 0.72), (iv) Total SoI ability score (PI 0.63) and (v) Playing football at school (PI 0.63). To confirm the optimal configuration of clusters, several iterations of the analysis validated the findings from this arrangement. Further, analyses included a split-half cross validation to assess internal consistency.

### Statistical methods

To determine differences between groups of children and the uniqueness of the clusters, univariate analyses, including independent *t-*tests (*t*), Pearson’s Chi-square test for association (χ^2^) and One-way ANOVA’s (*F*) were used. In relation to cluster membership, effect sizes were assessed using Cramer’s V coefficient and omega squared (ω^2^) to measure of the strength of the association. For all inferential tests, a *p* value of <.05 was taken to be statistically significant. All analyses were undertaken using IBM SPSS Statistics v24.

## Results

### Demographics

In total *N* = 594 schoolchildren completed the brief survey (60.2% from primary schools and 39.8% from secondary schools). For pupils providing data on gender (*n* = 577), 54.2% were boys and 45.8% were girls. The average age of participants was approximately 11 (±2.1) years old and there was no significant difference in age by gender. For primary school pupils, average age was 9.5 (±0.21) and average secondary school age was 13.4 (±1.56).

### Frequency of playing football

Children reported how often they played football inside and outside the school environment. Collectively, 40.9% of children reported playing football at least once a week at school, and 36.5% played football at least once a week outside of school. Conversely, 29.1% reported never playing football at school; 32.6% reported never playing football outside school. Moreover, there was a significant association between gender and frequency of playing football in (χ^2^ [4] = 86.722, *p* = 0.000) and outside school (χ^2^ [4] = 71.447, *p* = 0.000). Boys reported playing football significantly more often compared to girls. In total, 56.5% of boys played football at least once a week at school versus 22.3% of girls, and 49.8% of boys played football at least once a week outside school compared to 19.7% of girls. Further, 17.3% of boys reported never playing football at school versus 43.2% of girls, and 19.8% of boys never played football outside school compared to 47.3% of girls.

### Ego-net analysis

Overall, 60% of pupils reported having a personal network around football; the remaining pupils reported no footballing ego-network. There were significant associations between personal networks and playing football inside (χ^2^ [4] = 139.415, *p* = 0.000) and outside school (χ^2^ [4] = 185.517, *p* = 0.000). For pupils reporting a personal network (1 or more friends), 56.3% and 54.4% played football at least once a week at and outside school respectively, compared to 17.7% and 9.7% of pupils with no network. Further, there was a significant association between the presence of personal networks and gender (χ^2^ [1] = 30.097, *p* = 0.000). A larger proportion of boys (71.6%) reported a network engaged in football versus girls (49.2%). Data also revealed a significant association between personal networks and type of school (χ^2^ [1] = 65.118, *p* = 0.000). In primary schools, 73.8% of pupils reported football-oriented personal ties compared to 40.6% of secondary school pupils.

Table 1 summarises the ego network statistics. No significant differences were found between primary and secondary school pupils, or in network size, directed ties and ordered ties between boys and girls. However, compared to girls, boys reported a significantly greater network density (*t* [249] = 1.981, *p* = 0.049), significantly lower effective network size (*t* [249] = − 2.322, *p* = 0.018) and significantly lower network efficiency (*t* [249] = − 2.205, *p* = 0.028). Therefore, boys had a significantly greater proportion of ties that were actually present, or a denser network. In addition, due to a lower effective network size, boys had more alters and there were more ties between those alters. Therefore, information needed to pass to fewer people within boy’s networks to flow through it compared to girls. Further, boy’s network efficiency indicates a lower proportion of non-redundant ties. This suggests that boy’s networks have fewer structural holes, i.e. fewer gaps between friends, with complimentary sources of information around football. Further, boys (80.9%) accounted for a significantly larger proportion of nodes across all the networks (*t* [249] = 17.075, *p* = 0.000) compared to girls (19.1%). For boys, 97.5% of their network nodes were other boys, indicating a homophily effect around gender. Conversely, just 50.8% of girls’ network nodes were other girls, indicative of a heterophily effect. It is evident that network structure plays a complex role in football participation.

**Table 1:** Ego network summary

	Mean (±SD)				
	*All* *n = 251*	*Boys* *n = 165*	*Girls* *n = 86*	*Primary* *n = 176*	*Secondary* *n = 71*
Network Size	5.93 (±0.383)	5.92 (±0.433)	5.95 (±0.262)	5.94 (±0.388)	5.90 (±0.384)
Directed Ties	23.96 (±7.710)	24.52 (±7.679)	22.88 (±7.699)	23.83 (±7.972)	24.48 (±7.081)
Ordered Pairs	29.36 (±3.211)	29.26 (±3.549)	29.56 (±2.443)	29.47 (±3.100)	29.07 (±3.559)
Effective Network Size	2.00 (±1.187)	1.88 (±1.148)	2.25 (±1.230)	2.04 (±1.233)	1.88 (±1.048)
Network Density %	80.81	83.0	76.6	79.9	83.6
Network Efficiency %	34.1	32.0	38.0	34.6	32.1

Note: Primary = Primary school pupils, Secondary = Secondary school pupils

### Sources of influencer (SoI) questionnaire

Table 2 shows the pupils SoI scores. From a maximum of 60, the average score for all pupils was 40.8 (±13.19), this comprised scores of 19.9 (±7.25) for motivation and 20.9 (±6.42) for ability. Data indicated statistically significant differences in total SoI score by gender (*t* [554] = 8.616, *p* = 0.000), with boys reporting higher scores compared to girls. In addition, primary schools pupils reported significantly greater scores than their secondary school counterparts (*t* [551] = 4.133, *p* = 0.000). Pupils reporting personal networks engaged in football had significantly higher total SoI scores compared to pupils without such networks (*t* [565] = − 17.747, *p* = 0.000). Further, pupils with higher SoI totals played football more often in school (*F* [4, 562] =101.424, *p* = 0.000) and outside school (*F* [4, 562] =151.023, *p* = 0.000). Data showed a linear positive relationship between the regularity of playing football and SoI scores.

**Table 2:** Mean sources of influence (SoI) scores

Sources of Influence	All Pupil	Boys	Girls
*Personal Motivation*	6.7 (±2.80)	7.5 (±2.72)	5.8 (±2.53)
*Personal Ability*	7.1 (±2.22)	7.7 (±2.10)	6.3 (±2.13)
*Social Motivation*	6.8 (±2.52)	7.4 (±2.50)	6.0 (±2.40)
*Social Ability*	6.6 (±2.47)	7.4 (±2.40)	5.7 (±2.22)
*System Motivation*	6.5 (±2.56)	7.1 (±2.51)	5.7 (±2.40)
*System Ability*	7.2 (±2.44)	7.9 (±2.31)	6.4 (±2.40)
Total	40.8 (±13.19)	44.9 (±12.92)	35.8 (±11.78)

Note: The SoI sub-scales range from 0 to 10, maximum total score = 60

### Cluster analysis

In total, *n* = 567 pupils provided valid data for the cluster analysis. To maximise the similarity within, and variability between the participants, a three-cluster solution emerged. A split-half sample shaped and validated the result. The average silhouette - used to interpret and confirm solutions - was 0.4, representing a fair level of cohesion and separation. Further, the ratio between the largest and smallest clusters was 1.40, indicating that the clusters were of a similar size. Cluster 1 (*Traditional Market*) accounted for 27.7% (*n* = 157) of pupils, there were 33.5% (*n* = 190) pupils assigned to cluster 2 (*Sporadically Engaging Socialisers*) and 38.8% (*n* = 220) to cluster 3 (*Disconnected)*.

**Fig. 1:**
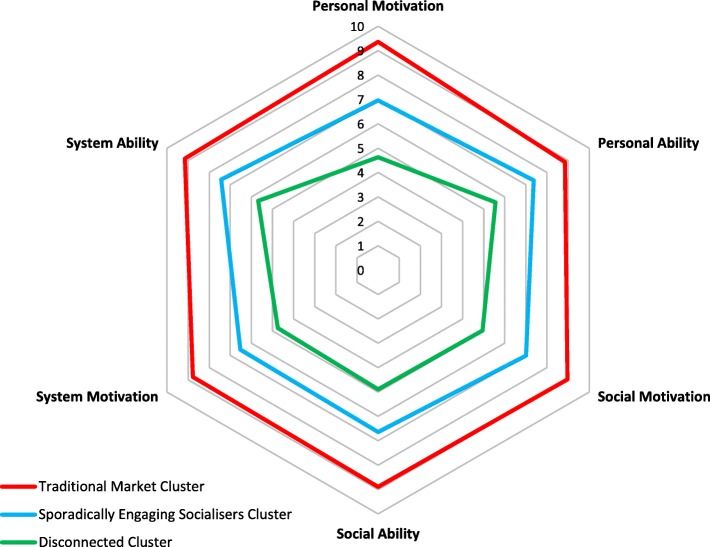
Cluster radar plot for the sources of influencer questionnaire

Table 3 shows the organisation of these clusters. The *Traditional Market* cluster was characterised by presenting footballing networks with no gendered differences, regular weekly football participation both inside and outside school and the highest scores on the SoI questionnaire for motivation and ability. The *Sporadically Engaging Socialisers* also presented personal footballing networks, yet, effective size and efficiency was significantly higher for girls compared to boys. Further, this cluster typically did not play football at school and rarely played football outside school. In addition, motivation and ability scores were lower - by 22% and 18.4% respectively - compared to the *Traditional Market* cluster. The *Disconnected* cluster had no personal networks engaged in football and did not play football inside or outside school. They also reported the lowest motivation and ability scores; respectively these were 20.5% and 17.8% lower than the *Sporadically Engaging Socialisers* cluster, and 42.5% and 35.9% lower than the *Traditional Market* cluster. Figure 1 shows the SoI scores for each cluster.

**Table 3:** Cluster characteristics

	*Cluster 1* *Traditional Market* *(n = 157, 27.7%)*	*Cluster 2* *Sporadically Engaging Socialisers* *(n = 190, 33.5%)*	*Cluster 3* *Disconnected* *(n = 220, 38.8%)*
*Social Network Around Football*	Have a football social network	Have a football social network	No social network around football
*Play Football Outside School*	Play at least once a week	Play at least once a month	Don’t play football outside school
*Total Motivation Score*	27.10 ± 3.085	20.49 ± 5.708	14.33 ± 5.697
*Total Ability Score*	26.89 ± 3.402	21.44 ± 4.900	16.13 ± 5.351
*Play Football at School*	Play at least once a week	Don’t play football at school	Don’t play football at school

Table 4 shows the different characteristics of the clusters. A significant association with a medium effect size was found between gender and cluster membership (χ^2^ [2] = 66.261, *p* = 000, *Cramer’s V* = .354). The *Traditional Market* cluster had twice the proportion of males compared to other clusters. There was a significant association with a medium effect size for cluster membership and school type (χ^2^ [2] = 57.425, *p* = 000, *Cramer’s V* = .326). Overall, the *Disconnected* cluster consisted of around 30% fewer primary school pupils, indicating that secondary schoolchildren were more likely to be disconnected, potentially making them harder to (re-)engage. There was a significant effect for cluster membership on the gender of alters (*F* [2, 564] = 209.578, *p* = 000, ω^2^ = .65). The Games-Howell post hoc test revealed that the *Traditional Market* cluster contained a significantly higher proportion of alters that were boys compared to the *Sporadically Engaging Socialisers* cluster (*p* = 000). Furthermore, the *Traditional Market* cluster had a significantly greater network density (*t* [239] = 2.420, *p* = 0.016) - 7.3%, significantly lower effective network size (*t* [239] = − 2.450, *p* = 0.015) - 0.37 - and network efficiency (*t* [239] = − 2.527, *p* = 0.012) - 6.5% - compared to the *Sporadically Engaging Socialisers* cluster. Indicating that more alters in the *Traditional Market* cluster send football related information to other alters; the information can reach other alters in the network more effectively as more ties are redundant, yet the network is less efficient as the proportion of non-redundant ties is lower.

**Table 4:** Between group differences in cluster features

	*Cluster 1* *Traditional Market*	*Cluster 2* *Sporadically Engaging Socialisers*	*Cluster 3* *Disconnected*	*Effect Size*
Gender (%)				*Cramer’s V* = .35
*Male*	81.7	47.1	40.7	
*Female*	18.3	52.9	59.3	
School Type *(%)*				*Cramer’s V* = .33
*Primary*	71.5	76.2	41.7	
*Secondary*	28.5	23.8	58.3	
Gender of Nodes *(%)*				*ω*^*2*^ = .65
*Boy Nodes*	88.1	73.2	0.0	
*Girl Nodes*	11.9	26.8	0.0	

## Discussion

To the best of our knowledge, this paper is the first to combine cluster with ego network analyses to point towards a better understanding of how sport-based physical activity initiatives can hinder some potential participants, yet succeed with others. One of our most important observations was how poorly this initiative performed in recruiting substantial numbers of children from either of two newly identified clusters. These under-recruited clusters featured (i) girls and (ii) children with insufficient footballing ego networks (a proxy for personal friendship networks); the targets of many so called inclusive sporting initiatives. This finding confirms previous research showing that stratified approaches to behaviour change across multiple layers of influence may prove beneficial [23]. The cluster analysis also showed that football was most effective at engaging already competent boys, motivated by football, who found it easy to participate where they lived and whose social networks focussed extensively on it. Their behaviour and social norms appeared to be reinforced by close football-oriented ties.

Unsurprisingly, social networks exerted a powerful influence on football participation. Simply having a football ego network was a significant moderator of participation; boys were more likely to report a footballing network compared to girls, and they engaged with football more regularly. Boys had significantly denser and more efficient networks; fewer non-redundant ties allowed a free-flow of information, and enhanced adoption of norms based around participation. Although gender did not significantly attenuate all structures of reported networks, boys’ alters were almost exclusively boys, a clear homophily effect. For girls, only half of their alters were other girls, limiting opportunities to bond and congregate with similar others around football. Therefore, in this instance, gender diversity influenced participation levels. Ultimately, addressing these gendered peer effects may help to enhance engagement [43]. Furthermore, networks based on homophily can become closed, making it difficult for outsiders to reach the inner core and adopt the prominent norms the group adhere too, which may generate social isolation and feelings of anomie. To improve recruitment of traditionally unresponsive groups, like girls and the many boys outside the *Traditional Market* cluster, creating denser football-based networks will help make interactions around football both easy and inevitable. In essence, practitioners need to adopt needs-led bottom up approaches that actively listen to these groups and engender networks that represent and reflect them. Paradoxically, the best way to engage the non-participating groups (identified in the *Sporadically Engaging Socialisers* and the *Disconnected* clusters) in existing provision would be for them to share more characteristics of the *Traditional Market* cluster.

With just three clusters, homophily was powerfully evident in this study. Consistent with previous research [44], respondents clustered and had additional frequent contact with similar others. The large number of structural holes, or less dense networks, seen in the *Sporadically Engaging Socialisers* cluster was associated with lower levels of participation, and low levels of motivation to engage with, and ability to play football. In this instance, greater cohesion through denser ties equated to improved participation levels. These sparse social networks made it difficult to find and take advantage of opportunities more easily realised through the denser networks of individuals in the *Traditional Market* cluster. Further, children and young people in the *Traditional Market* cluster benefited from their more densely connected network, by developing their footballing competence; this had concomitant effects on their motivation. It is likely this acts as a virtuous cycle to increase further engagement.

With new pressures on widening participation, it may be surprising that policy makers and practitioners address their current battles as they addressed previous ones. The assumption that conventional approaches will reach new audiences and grow the game are likely misguided [45]. Like iron filings attracted to a magnet, the *Traditional Market* cluster identified in this study naturally gravitated to football. The standard football offer clearly attracts this group. Yet, to attract the *Disconnected* or *Sporadically Engaging Socialisers* clusters, practitioners should not assume that the problem of (non)-participation lies with the intended recipients. In reality, different mediums for magnetism are needed to recruit participants from the *Sporadically Engaging Socialisers* and *Disconnected* clusters. For them, football investment may have been more influential by actively creating powerful positive social influences and offering strong structural signalling, before and/or alongside a focus on footballing ability and motivation.

### Strengths and limitations

Our findings should be viewed in light of the studies methodological strengths and limitations. First, the external validity of the results may be limited due to volunteer bias resulting from the non-probability sample and sample size. This limits the generalisability of the findings to other youth sports, although similar clusters and influences for (non) participation may exist. Further, although the evidence of between cluster differences, networks and participation was strong, establishing causality is harder to identify in any cross-sectional study design [23]. Given our reliance on self-report, results reflect the pupils’ own understanding of their status rather than objective assessment. Response bias and an unknown level of ascertainment bias may have also affected survey responses. Study strengths include the novel approach and new insights generated by the ego network analysis regarding the structure of pupils’ networks around football. Further, the cluster analysis revealed tangible between-cluster heterogeneity and within-cluster homogeneity; this supports the underlying notion of concurrent influences affecting (non) recruitment into this programme.

## Conclusion

This study provides new insights into one of the most poorly understood questions facing physical activity and sporting systems, how to increase participation among non-engaging groups of children. Using clustering and network analysis, underpinned by a behavioural systems framework, we have identified key sources of influence currently working for and against this goal. Our study strongly suggests that individuals attach to macro structures, like football, directed by local connections among personal networks. Further, the clustering identified within this system points towards undertaking campaigns that not only target groups but also how their living environments can be better managed to support easy engagement and successful participation. Moreover, having unpicked these behavioural influences and identified how they support large clusters of children, practitioners can now concurrently deploy these powerful sources of influence to make positive change more likely.

## Abbreviations

BAME: Black and Minority Ethnic
CoF: City of football
PI: Predictor Importance
SoI: Sources of influence
